# RETRACTED: Maifata et al. Role of Serum and Urine Biomarkers (PLA2R and THSD7A) in Diagnosis, Monitoring and Prognostication of Primary Membranous Glomerulonephritis. *Biomolecules* 2020, *10*, 319

**DOI:** 10.3390/biom13121710

**Published:** 2023-11-27

**Authors:** Biomolecules Editorial Office

**Affiliations:** MDPI, St. Alban-Anlage 66, 4052 Basel, Switzerland; biomolecules@mdpi.com

It has come to our attention that an error occurred in the “Methods and Materials” section of this manuscript [1]. In the published paper, the authors did not mention clearly if they checked for the PLA2R1 antigen or for the antibodies against this antigen. The authors were unable to clarify and ascertain this point, and as this point causes confusion to the readers and might affect the conclusions of the entire study, it has been decided to retract the paper. The published paper [1] will therefore be marked as retracted.

The retraction of the article was recommended by an expert Editorial Board Member and approved by the Editor-in-Chief of *Biomolecules*. MDPI is a member of the Committee on Publication Ethics and takes seriously its responsibility to publish only high-quality research. We regret that these issues were not identified earlier and apologize to readers of the journal. We note that the retraction is not supported by the authors, who insisted that the original results still stand.
